# It’s Not Just about Bicycle Riding: Sensory-Motor, Social and Emotional Benefits for Children with and without Developmental Disabilities

**DOI:** 10.3390/children9081224

**Published:** 2022-08-13

**Authors:** Sarah A. Schoen, Vincentia Ferrari, Andrea Valdez

**Affiliations:** 1 Research Department, STAR Institute, Centennial, CO 80112, USA; 2 Research Department, Creighton University, Omaha, NE 68178, USA

**Keywords:** sensory-motor therapy, sensory processing and integration, motor coordination, social participation, developmental disabilities

## Abstract

Background: A developing area for therapy is teaching children to ride a bicycle. Little has been written about the effectiveness of these programs. This study explored outcomes from participation in a novel bicycle riding program for children with a wide array of developmental challenges. Method: Two studies were conducted; a nonconcurrent, multiple baseline design with four participants and a pretest–posttest single group with 15 children. Results: Study 1 participants improved on broad jump. Balance positions showed variable responses. Study 2 participants showed significant improvement on broad jump, and a trend toward significance walking forwards and backwards. Parents reported improvement in following rules, participating in daily routines, interacting with peers, and feeling good about him/herself and a change in child’s participation in community and extracurricular activities. All children improved in glide time or achieved independent riding. Conclusions: Preliminary evidence was found for the effectiveness of one approach for developing motor and social skills within the context of learning to ride a bicycle. Children over the age of 6 years were able to ride a two-wheeled bicycle at the end of the program. Participation suggested improvement in motor coordination and dynamic balance as well as changes in measures of social interaction and self-esteem.

## 1. Introduction

Physical activity is central to health and well-being [1], however, there has been an increasing reduction in children’s participation in physical activities due to involvement in screen time [2]. In particular, children with disabilities are at a greater risk of avoiding physical activity due to motor challenges that make it difficult for them to keep up with their peers [3].

Learning to ride a bicycle is a keystone in a child’s development as well as being a popular form of recreation and physical activity for children [4]. Bicycles are central in the social and physical lives of most children and provide a mechanism through which positive peer relationships develop [5,6]. Not only is bicycle riding important in the development of motor coordination, strength, and endurance, but it is also a common form of physical activity that supports a child’s development of self-esteem [7]. Research addresses the importance of engaging children with disabilities in physical activity to prevent further inactivity [8], so bicycle riding can offer an accessible and relatively inexpensive pleasurable, independent form of physical activity. However, for children with disabilities, this goal may take longer, be more arduous, and may not be achievable [9,10]. Motor skill challenges can impair performance and self-esteem issues can reduce motivation for participation and learning [6].

A developing area for therapy practices is teaching children to ride a bicycle [11]. It has been identified as a meaningful occupation of childhood and is often identified as a therapeutic goal for children and families. However, traditional methods of teaching bicycle riding may not be effective for children with disabilities due to delays in balance, motor coordination, fear, and lack of self-esteem [9]. Additionally, other therapeutic goals related to daily functioning may take priority, so bicycle riding may not be included in individual treatment sessions. Lastly, parents may not have the knowledge and skill to know how to best teach their child how to ride a bicycle. 

Learning to ride a bicycle lends itself to group training models that are increasingly more popular in rehabilitation settings and can be delivered in a clinic or school. Groups provide an opportunity for peer support and peer modeling [12]. The support of the group, and the shared goal, can also provide increased motivation. Literature suggests that children can benefit from learning from other children in the form of a group [13].

Several bicycle riding training programs exist, showing moderate to strong success rates. Two studies of youth and adolescents with Down syndrome or autism report a high success rate in learning how to ride after a five-day training program lasting 75 min per day [5,10]. Another study provides evidence of 100% success in learning to ride a bicycle following a program that combined group and individual session over five days [14]. Children with Down syndrome reported a slightly lower success rate (e.g., 56%) but showed bike riding was related to significantly less time spent in sedentary activity 12 months post intervention [15]. Lastly, a study of children with developmental coordination disorder had a success rate of 89% with a range of training time from 2 to 16 h. All studies concluded that mastering bicycle skills is an important developmental milestone that contributes to a child’s happiness [14] and engagement in meaningful activity [10], however, none of these studies included measures that reflected changes in social interaction, relationships with peers/family, or feelings of self-esteem.

This pilot study reports on a unique bicycle riding program that employs specialized equipment and innovative teaching techniques designed to address the needs of a wide array of disabilities or challenges. While the focus of the group is on learning to ride a bicycle, the group also focuses on shared joy and social interaction amongst peers. Importantly this study was designed to identify outcomes that were sensitive to change in the social/emotional domain along with gains in sensory-motor processing. 

Thus, the following research questions were posed:

1. What sensory-motor outcomes are sensitive to change and reflect the benefits of participation in an adapted bicycle group intervention for children with sensory and developmental challenges?
2. What social/emotional outcomes are sensitive to change and reflect the benefits of participation in an adapted bicycle group intervention for children with sensory and developmental challenges?

A single-subject research design was utilized to pilot procedures, assess feasibility of the methodology, as well as identify sensitive outcome measures. This was followed by a small single group pretest–posttest study.

## 2. Materials and Methods

Two studies were conducted to pilot procedures and identify sensitive outcome measures that investigate a bicycle intervention implemented over five consecutive days to promote the ability of children to ride a two-wheel bicycle. The first was a nonconcurrent, multiple baseline design with one participant from each of four consecutive sessions that served to investigate the sensitivity to change of the outcome measures. The second study was a small pretest–posttest single group design that combined data from summer groups offered over two consecutive years.

### 2.1. Participants 

Participants were recruited from multiple sources, from the clinic at which the program was run, from a community agency supporting children and families with developmental disabilities, as well as from email solicitations and word of mouth. Inclusion criteria were based on the child’s inability to ride a two-wheel bicycle independently due to sensory, motor, or social-emotional challenges, and willingness of the child to wear a helmet. These were children who had not been successful learning to ride a bicycle and wanted to learn to ride. Additionally, children had to be ambulatory, have the ability to use their upper and lower limbs to steer and propel a bicycle, and were cognitively capable of understanding the focus/nature of the group. Children were excluded if they were under 4 years of age or if they had a significant motor or intellectual impairment such as cerebral palsy, Down syndrome or intellectual disability. 

For Study 1, children were excluded if they could not complete the required visits for the baseline and intervention phase.

#### 2.1.1. Study 1

Four children participated in the pilot nonconcurrent, multiple baseline study, 1 male and 3 females ranging in age from 7.91 years to 9.71 years (M = 8.95). Each child was enrolled from a separate but consecutive group session. One female had a diagnosis of attention deficit hyperactivity disorder (ADHD), one female had a diagnosis of developmental delay, and the other female had no formal diagnosis. The male had a diagnosis of autism. Sensory processing and integration challenges were obtained through parent report using the Sensory Processing 3 Dimensions (SP3D) Inventory. Posture or praxis challenges were reported in two of the four participants.

#### 2.1.2. Study 2

Fifteen children participated in the pretest–posttest study, of data collected over two years from four different groups. 11 males and 4 females ranging in age from 4.43 to 10.6 (M = 6.49). All participants had previously identified sensory processing and integration challenges, six with the following comorbid diagnoses: developmental delay (*n* = 4), autism (*n* = 1), and ADHD (*n* = 1). Sensory over-responsivity was reported on the SP3D Inventory in 80% of the participants and posture or praxis challenges in 60%. Sixty-seven percent of those with sensory over-responsivity (SOR), also had either posture or praxis challenges. 

### 2.2. Procedures

#### 2.2.1. Study 1 

Pre-intervention data was collected from all participants via an intake phone call during which the group leader was able to determine the appropriateness of the child for enrollment in the group. 

Procedures were approved by the Institutional Review Board of Rocky Mountain University of Health Professions. Informed consent was obtained from all parents of the participants and assent was obtained from all participants over the age of 7.

All participants attended daily one-hour group sessions for one week (e.g., five consecutive days). None of the children had previous experience with this bicycle training program. Study 1 was designed to have four baseline and five intervention probes (e.g., one each day). Length of actual baseline and intervention phase data varied by participant attendance. 

Following Study 1, the parents of participants were interviewed in order to obtain information about their perception of other motor or social-emotional outcomes and their satisfaction with the intervention. Modifications were made in Study 2 to more systematically collect feedback from parents by the inclusion of a parent report Visual Analog Scale of common gains reported in Study 1 as well as the Occupational Performance Scale of the Sensory Processing Three Dimensions Measure (SP3D). 

Once enrolled in the group, baseline data was collected the week before the first day of the bicycle intervention. Subsequent intervention data were collected daily over the course of the five-day program.

#### 2.2.2. Study 2

Study 2 included pre-intervention and post-intervention administration of the sensory and motor measures from Study 1. Due to the variability amongst the four participants, all measures were included. Additionally, parent reports on the SP3D Occupational Performance Scale and the Visual Analog Scale questions were obtained pre and post-group participation. 

Once enrolled in the group, child participants were administered the sensory-motor activities on the first day of the group 30 min prior to the start of the group session and then again after the final session on Day 5. Parents of the participants completed the caregiver report measures while children participated in the first session and then at the end of the week.

### 2.3. Measures

#### 2.3.1. Outcomes 

The sensory-motor outcomes for Study 1 and Study 2 were selected to assess balance, strength, sensory processing, and motor coordination. These postural components were selected based on research that supports the importance of motor skill development as contributing to success in bicycle riding, citing strength, balance, coordination and endurance [6,16,17]. These activities were subtests from the Sensory Processing Three Dimensions (SP3D) Measure [18]. Research assistants were trained in administration by the authors of the scale. Activities are described as follows:

- *Standing Broad Jump* is a measure of leg strength [19] that reflects the child’s ability to jump forward with two feet. This activity is scored for distance to the nearest inch.
- *Reach and Rock* is a measure of vestibular over-responsivity [20] and reflects the child’s performance of a five-step movement sequence: reaching down to touch the back of their ankles, followed by reaching back to touch the wall behind. Scoring criteria is based on child’s capacity to complete the task of tilting their head forward or backward in space. (Avoidance = 1, no avoidance = 0).
- *Statue Game* is a measure of static balance [21]. In two positions and two conditions, each with eyes open and eyes closed: (1) two foot standing position on the floor, (2) two-foot standing position on a balance pad, (3) one-foot standing on the floor and (4) one-foot standing on a balance pad. Each position is scored for time up to 10 s.
- *Heel-Toe Walking Forward* is a measure of dynamic balance [22] that requires the child to walk forward using a heel-to-toe gait along a 12-foot-long, 1-inch thick line. Scoring is based on the number of accurate heel-toe steps completed within 10 s (heel and toe within 1 inch of each other).
- *Walking Backwards* is also a measure of dynamic balance and coordination [23,24] that requires the child to walk backwards along the same 12-foot-long line using a regular gait. Scoring is based on the number of accurate steps completed within 10 s (e.g., the majority of the foot remaining on the line).

All activities were scored from videotape recordings by two independent research assistants (RAs). Video recordings were randomized so that RAs would be blind to the session number. Inter-rater reliability calculations at three different time points were 88%, 96%, and 100%.

#### 2.3.2. Parent-Report Measures 

In Study 1, parents were interviewed in order to obtain feedback on the social-emotional gains experienced by their children. In Study 2, parents completed the SP3D Occupational Performance Scale (OPS) [18], as well as the Visual Analog Scale (VAS) questionnaire developed by the authors based on feedback provided by parents in Study 1. The SP3D Inventory was used to characterize the sensory processing and integration challenges of participants in both studies.

The Sensory Processing 3 Dimensions Scale (SP3D) [18] is a newly developed measure of sensory processing and integration abilities. It includes a performance assessment and two parent report scales that are part of a comprehensive occupational therapy assessment of children with sensory processing and integration challenges. The SP3D OPS measures participation in daily life across five categories of function: Relationships, Ability to follow routines at home, Ability to perform activities of daily living, Performance at school or in the classroom, and Participation in Community and Extracurricular activities. Questions are rated from Poor (1) to Excellent (4) based on the degree of parent perceived competency.

The SP3D Inventory measures atypical sensory-related behaviors associated with sensory over-responsivity, sensory under-responsivity, sensory discrimination, posture, and praxis. Preliminary data shows good reliability and validity [25,26].

The Visual Analog Scale (VAS) taps behaviors related to social, emotional, and motor participation outside of the group. The VAS is presented as a 10-centimeter horizontal line that is anchored by two verbal descriptors, never and always. There are no numbers or additional verbal descriptors on the scale, which minimizes scoring bias around a preferred numeric value. Parents are asked to make a slash mark anywhere between the endpoints of the line to rate their response to the question. Each question is scored from 0 to 10 based on where the parent makes a slash mark.

#### 2.3.3. Riding Proficiency 

Riding proficiency was measured in two ways: glide distance (based on time) and independent riding. Glide distance was defined as the time the child was able to propel him/herself forward while seated on the bicycle with feet off the ground. Glide times were collected on each participant who did not achieve independent riding pre and post the bicycle training program. Independent riding was defined as the ability to balance, steer, and pedal a two-wheeled bicycle for a minimum of 3 full foot/pedal rotations, which is consistent with previous literature that suggests a distance of 30 or more feet is the minimum distance needed for independent riding [9].

### 2.4. Setting and Equipment

The intervention took place at a facility that had a large outdoor playground with a track around the perimeter. The final day was in an open outdoor space adjacent to a parking lot.

Strider balance bikes were used. These lightweight, two-wheeled bicycles have a slightly lower center of gravity and no pedals. Children learn to glide by pushing with their feet and then lifting them off the ground; stopping is achieved by putting their feet on the ground.

### 2.5. Description of the Intervention 

The training protocol and instructional techniques for this intervention were developed by the STAR Institute and are based on the STAR Frame of Reference manualized approach for groups [27]. Instructional techniques were adapted from a series of recommended steps for teaching a child to ride on the Strider Sports International website. Online training is available on their website (All Kids Bikes Inclusive Programs. Available on line https://www.striderbikes.com/learning/#instructor accessed on 7 July 2022). Group sessions include the following content: body warm-up, balancing activities, handling bikes, mastering gliding, follow the leader, making turns, weaving through cones, starting and stopping, and obstacle course navigation (see Supplemental Materials, File S1 for Description of the Curriculum).

All participants were required to wear a bicycle helmet anytime they were on the bikes. Each training session consisted of five or six participants. Groups were led by an Occupational Therapist and three to four assistants (OT students, interns, volunteers). Each group was individually tailored to the level of proficiency and needs of the child participants and one-to-one attention was offered when needed, such as shortening obstacle course navigation, providing external support for balance activities, etc.

The emphasis of the group was threefold: (1) children learning to ride a bicycle, (2) children experiencing joy and feeling good about themselves within the context of being on a bicycle, and (3) children sharing a positive experience with peers. Guiding principles included connecting with attunement, fostering success, maintaining arousal regulation, and facilitating circles of communication. These components of the STAR Frame of Reference [27] were incorporated into each group session and implemented to support joy, self-esteem, and motivation to participate. Fidelity to the manualized approach was ensured via video coding or live observation for at least one day in each session. 

### 2.6. Interventionist

The interventionist for this study was an occupational therapist with 12 years of experience in pediatrics. She has Proficiency Certification Level 1 in sensory processing and integration challenges and completion of Level 2 Mentorship at the STAR Institute, as well as Level 1 certification in DIR/Floortime [28]. 

### 2.7. Data Analysis

#### 2.7.1. Study 1

Data is presented in Supplemental Materials. Data analyses followed recommendations by Kennedy ([29] 2005). Visual analysis of change in mean level, trend (e.g., rate of change), and percent of non-overlapping data were used to analyze the data. The mean level was calculated by averaging data points within the baseline condition compared to the treatment condition. See Table S1. The trend was evaluated using the size and direction of the slope. Changes in the mean level and trend in performance were examined for the sensory-motor probes. Percent non-overlapping data (PND) represents the data points during the intervention phase that do not overlap with the baseline data points [30]. The higher the PND, the stronger the support for a treatment effect [31]. Study 1 data was used to evaluate the feasibility of the methodology and the sensitivity of the outcome measures. See Figures S1–S4.

#### 2.7.2. Study 2

In Study 2, all pre–post treatment measures were evaluated using the Wilcoxon signed-rank test. No correction was made for multiple comparisons due to the pilot nature of this study. The sensory-motor activities were videotaped and coded by a research assistant who was blind to session number. The VAS was scored by computing the point on the line marked by the parent from ‘0’, a score of the behavior never occurring on the far left of the scale, to ‘10’, indicating behavior was always present during the preceding week. 

Clinical data were collected from all participants in Study 1 and Study 2 regarding riding proficiency after one week of training. If the participant did not achieve independent riding, glide distance pre and post intervention was reported.

## 3. Results

Demographics for all participants appear in Table 1.

All procedures for Study 1 were completed successfully. The sensory-motor activities selected for Study 1 to measure the effect of bicycle riding group intervention produced relatively positive results (see Supplemental Materials Figures S1–S4 and Table S1 for results). 

All of the participants improved in Broad Jump and Reach and Rock. Statue, Heel Toe Walking Forwards and Backwards Walking did not show consistent change. Statue Game balance positions showed the most variable responses across six conditions, although each participant improved in at least one balance measure. 

In Heel Toe Walking, three participants showed positive change. In Backwards Walking, two participants showed positive change. Mean level and percent non-overlapping data appear in the Figures S1–S4 in Supplemental Materials. Due to the pilot nature of this study and the variability of the findings, all sensory-motor measures were included in Study 2. All participants met the criteria for independent riders.

In Study 2, sensory-motor outcomes were collected on 15 participants and showed mixed results. On these activities, there was a significant improvement on Broad Jump, and a trend toward significance in Heel Toe Forward and Backwards Walking. Reach and Rock and duration of Balance across conditions were unchanged (see Table 2).

Only 14 parents returned the parent report measures. Outcomes on the VAS showed statistically significant improvement from pretest to posttest on four of the seven responses. Parents reported improvement in following rules, participating in daily routines, interacting with peers, and feeling good about him/herself. The other three responses (e.g., confidence in movement activities, playful, or kind to family, and staying calm during disagreements) were not significant but changed in the expected direction (see Table 3).

There was a trend toward significant change in the Play and Extracurricular Activity subtest of the SP3D OPS (see Table 4).

Eight participants in Study 2 improved in glide distance and six participants met the criteria as independent riders following participation in the training (see Table 5).

## 4. Discussion

This study contributes to the methodology for studying the outcomes from a bicycle riding program and provides preliminary evidence regarding the positive impact of one approach for developing motor and social skills within the context of learning to ride a bicycle. This pilot study had two parts: Study 1 demonstrated the feasibility of the procedures but showed mixed sensitivity of outcome measures, thus all measures in Study 1 were included in Study 2 (e.g., strength as measured by Broad Jump, balance and coordination as measured by Balance, Walking Forwards and backwards). Parent interviews suggested the inclusion of a VAS to capture improvements in social and emotional functioning. Participation in the small group pretest–posttest study (e.g., Study 2) suggested improvement in motor coordination and dynamic balance as well as changes in measures of social interaction (interacted with peers), participation in daily routines, and self-esteem (felt good about self). In Study 1, 100% of the children achieved independent riding, successfully pedaling a two-wheeled bicycle for over three full foot/pedal rotations by the end of the five-day intervention. In Study 2, eight of the children improved in glide duration, and six of the participants achieved the goal of independent riding.

Interestingly, static balance (e.g., Statue Game) measures were a less meaningful outcome measure than measures of strength (e.g., Broad Jump). This is consistent with research supporting the association between learning to ride a bicycle and increased leg strength [32]. Specifically, MacDonald [10] found that youth with disabilities who learned to ride had greater leg strength than individuals who did not learn to ride. In this study, an improvement in leg strength was evident just by participating in this training program, whether they achieved independent riding or only improved in glide distance. Additionally, it is possible that the adapted bicycle technology of the Strider bikes helped riders achieve dynamic balance within the context of the riding activity, even though this change did not generalize to the static standing activities tested.

Unexpectedly, the measure of vestibular responsivity was sensitive to change in Study 1 but not sensitive to change in Study 2. The movement sequence performed in the Reach and Rock task typically elicited an adverse reaction in children who have vestibular over-responsivity. Clinically, occupational therapists have associated vestibular over-responsivity with difficulty achieving motor skills that involve movement of the head/body through space [20,33]. As the results of Study 2 suggest, difficulty riding a bicycle may have had more to do with strength and dynamic balance than an adverse reaction to linear movement. Further research is clearly needed.

Unique to this study was the inclusion of social and emotional outcome measures not reported or systematically studied in previous studies. Study 2 showed parent-reported improvements in children’s self-esteem as well as in social interaction with peers, participation in daily routines, and ability to follow the rules. Although not significant, there was a positive change in the direction of greater participation in community and extracurricular activities. While acknowledged in other research that success in bicycle riding improves motivation as well as producing positive health benefits [10,34], this study provides evidence of quantifiable changes in only one week of intervention. These behaviors are foundational to future motor and social challenges the child may experience in other settings.

The finding that only six of the children in Study 2 achieved independent riding was also surprising. One possible explanation is that half of these children were only 5 years of age or younger, so it is possible that the goal of independent riding was more difficult for them to achieve in a weeklong program. Notably, all of the younger children still improved in bicycle riding skills, as evidenced by increases in glide distance. For those younger children, a longer program may have resulted in a higher independent riding rate. Thus, findings from this study partially support previous research; successful bicycle riding can be achieved in five days in children with developmental challenges who are 6 years and older. This is consistent with studies showing children learned to ride using a variety of dosage models: 2 h/4 days a week [11]; 75 min/5 days a week [15]; 45 min sessions 3 times a week in a group and 2 individual sessions [14]. This study provided intervention for only 60 min a day for five consecutive days in a group format without the need for any individual sessions. However, it is possible that an extended program might produce even stronger results, especially for younger children or those with greater impairments.

Trends that were not statistically significant but still important outcomes for this study included improvement in dynamic balance, as reflected in walking forwards and walking backwards, as well as changes in participation in play and extracurricular activities on the occupational performance scale. These findings suggest that although static balance measures did not consistently improve, dynamic balance may be more closely associated with balance needed to learn bicycle riding. Additionally, a socially and emotionally positive bicycle riding experience, combined with physical success, may have provided motivation for these children to want to participate in other leisure activities they otherwise would not have attempted. Thus, greater opportunities for participation within the community were afforded them.

Like similar research focused on teaching children with motor impairments how to ride, this study advocates for the use of an adapted bicycle that addresses the balance and coordination difficulties of children with special needs [9]. The Strider balance bike used in this study appears to be an appropriate learning tool for these participants. The mechanics of this bicycle allowed children to experience the dynamics of riding under more supportive conditions.

Bicycle riding is an important form of physical activity that can be accessed by children with developmental challenges. Group programs are adaptable to school or clinic settings [10] and can support both social-emotional and motor aspects of development. The lack of adequate physical activity is a societal problem and a problem for children with special needs in particular [10]. Physical activity sets the stage for social engagement, self-esteem, and motivation to engage in new challenges [14,35]. Riding a bicycle, in particular, also brings with it a special opportunity for cultural/family acceptance amongst one’s peers [34].

## 5. Limitations

This study had several limitations. The first limitation was that the study took place at one private clinic location and, therefore, the results are not generalizable to other geographic regions. Additionally, parents who volunteered to allow their children to participate were primarily Caucasian and of similar parental education levels. The time between referral and group participation did not allow for the administration of a comprehensive occupational therapy evaluation and, therefore, information related to their sensory and motor challenges was gathered through medical and history forms. Furthermore, there was a lack of a control group, and although success was achieved within the five-day intervention, participants were not followed after completion of the group to determine if riding skills were maintained. Future studies should add to the methodological rigor as well as including a planned follow-up with participants to determine what percentage were able to maintain their riding ability.

## 6. Conclusions

In conclusion, the results of this pilot study provide preliminary support for the feasibility and sensitivity of outcome measures and suggest that participation in the STAR bicycle riding training program leads to both the acquisition of conventional cycling skills, as well as aspects of social and emotional functioning in preschool and school-aged children. The majority of the children over the age of 6 years were able to ride a two-wheeled bicycle at the end of the program. Parents reported gains in social participation, self-esteem, relationships with peers and family, as well as increased participation in daily life activities. Measures of strength and dynamic balance supported these outcomes and likely impacted children’s motivation to participate in other community-based extracurricular activities.

## Figures and Tables

**Table 1:** Study 1 and 2 Demographics.

	Study 1	Study 2
	*n* = 4	*n* = 15
Characteristics	(3 F, 1 M)	(4 F, 11 M)
Age (years)	8.95 (7.91–9.71)	6.48 (4.43–10.6)
Ethnicity		
Caucasians	4	14
Mixed		1
Maternal Education Level		
High School		1
College	2	5
Post-Graduate	2	9
Paternal Education Level *		
High School		3
College	2	5
Post-Graduate	2	6

*Note:* M = male; F = Female. * Single-parent household, no data on one participant’s paternal education level in Study 2.

**Table 2:** Study 2, Pre and Post Balance, and Walking Tasks, *n* = 15.

Task	Pre	Post	Wilcoxon		Eta-Squared
	M (SD)	M (SD)	*Z*	*p*	*η* ^2^
Standing Broad Jump (inches)	34.23 (12.13)	38.73 (10.57)	−2.54	0.011 *	0.655
Reach and Rock (# completed)	2.86 (2.07)	2.93 (2.30)	−0.686	0.493	0.177
Two Feet, Pad, Eyes Open (s)	10.00 (0)	9.80 (.780)	1.00	0.317	0.258
Two Feet, Pad, Eyes Closed (s)	9.07 (2.09)	9.00 (2.80)	−0.135	0.893	0.035
Romberg, Floor, Eyes Open (s)	6.80 (3.47)	7.73 (3.24)	−0.912	0.362	0.236
Romberg, Floor, Eyes Closed (s)	4.00 (3.51)	4.53 (3.07)	−0.696	0.487	0.180
Romberg, Pad, Eyes Open (s)	5.67 (4.12)	7.07 (3.28)	−1.53	0.125	0.396
One Foot, Floor, Eyes Open (s)	6.87 (3.14)	6.47 (2.72)	−0.434	0.664	0.112
One Foot, Floor, Eyes Closed (s)	2.93 (2.43)	3.53 (2.75)	−0.528	0.597	0.136
One Foot, Pad, Eyes Open (s)	5.60 (3.80)	5.47 (3.50)	−0.277	0.782	0.072
One Foot, Pad, Eyes Closed (s)	1.73 (1.34)	1.93 (1.53)	−0.578	0.563	0.149
Heel-Toe Walk Forward (# steps)	8.13 (5.57)	9.80 (5.55)	−1.67	0.095 †	0.430
Backward Walking (# steps)	5.87 (3.91)	7.00 (3.25)	−1.85	0.065 †	0.477

* Significant at 0.05 level; *Note*: s = seconds; # = number; † Trending at <0.10 level.

**Table 3:** Study 2, Pre and Post Visual Analog Scale, *n* = 14.

Question	Pre	Post	Wilcoxon		Eta-Squared
	M (SD)	M (SD)	*Z*	*p*	*η* ^2^
My child was confident in movement activities.	6.08 (2.48)	7.46 (1.48)	−1.66	0.096	0.445
2. My child followed the rules when playing games with others.	5.45 (1.92)	6.99 (1.49)	−2.36	0.019 *	0.629
3. My child was successful participating in daily routines.	5.49 (1.66)	7.19 (1.57)	−2.64	0.008 **	0.705
4. My child made friends and interacted well with peers.	6.29 (2.00)	7.82 (1.00)	−2.20	0.028 *	0.587
5. My child was playful or kind when engaged with other family members.	6.19 (1.56)	7.09 (1.79)	−1.51	0.131	0.403
6. My child seemed to feel good about him/herself.	6.21 (1.69)	7.63 (1.43)	−2.61	0.009 **	0.696
7. My child stayed calm when there were disagreements.	3.57 (2.09)	4.47 (2.34)	−1.48	0.140	0.395

* Significant at 0.05; ** Significant at 0.001.

**Table 4:** Study 2, Pre and Post SP3D Occupational Performance Scale, *n* = 14.

	*n* of	Pre	Post	Wilcoxon		Eta-Squared
	Items	M (SD)	M (SD)	*Z*	*p*	*η* ^2^
OPS: Category						
Relationship	2	9.00 (1.24)	9.07 (1.27)	−0.277	0.782	0.074
Routines at Home	2	7.64 (1.91)	7.86 (1.17)	−0.552	0.581	0.148
Activities of Daily Living	2	8.43 (2.21)	8.79 (1.42)	−0.785	0.432	0.210
School Activities	2	8.93 (1.64)	9.21 (1.72)	−1.10	0.271	0.294
Play/Extracurricular	3	12.31 (2.25)	13.29 (1.98)	−1.38	0.166 †	0.370

† Trending at <0.18 level.

**Table 5:** Study 2: Pre and Post Glide Times, *n* = 14.

Age	Gender	Diagnosis	Glide Pre	Glide Post
4.43	M	Developmental Delay, Sensory Integration, and Processing Challenges	4	16
4.61	M	Sensory Integration and Processing Challenges	0	5
4.73	M	Sensory Integration and Processing Challenges	5	16
5.04	M	Developmental Delay, Sensory Integration, and Processing Challenges	0	Riding
5.75	F	Sensory Integration and Processing Challenges	3	Riding
5.86	M	Sensory Integration and Processing Challenges	0	13
5.95	F	Developmental Delay, Sensory Integration, and Processing Challenges	1	14
6.51	M	Sensory Integration and Processing Challenges	5	Riding
6.61	M	Autism, Developmental Delay, Sensory Integration, and Processing Challenges	1	8
6.97	M	ADHD, Anxiety, Sensory Integration, and Processing Challenges	4	Riding
7.09	M	Dyspraxia, Sensory Integration, and Processing Challenges	1	Riding
8.16	M	Sensory Integration and Processing Challenges	2	16
8.55	M	Sensory Integration and Processing Challenges	2	Riding
10.6	F	Developmental, Cognitive Impairment, Language Delay, Sensory Integration, and Processing Challenges	0	2

## Data Availability

Data supporting reported results can be found on the STAR Instittute’s password protected server. The data presented in this study are available on request from the corresponding author. The data are not publicly available due to restrictions, e.g., privacy or ethical concerns.

## Supplementary Materials

The following supporting information can be downloaded at: https://www.mdpi.com/article/10.3390/children9081224/s1, Table S1: Study 1, Movement activities, mean level, and slope; Figures S1–S4: Participant multiple baseline graph; File S1: Description of the program.
