# Supplementary material for: It’s Not Just about Bicycle Riding: Sensory-Motor, Social and Emotional Benefits for Children with and without Developmental Disabilities
Source: Children (Basel). 2022 Aug 13;9(8):1224. doi: 10.3390/children9081224 (PMC9406935; doi:10.3390/children9081224)
Supplement: Supplementary file 1 [file children-09-01224-s001.zip › File S1.pdf]

File S1. BIKE CAMP Curriculum

	Activity	Resources
DAY 1	1. Body warm up<br>• Head shoulders knees and toes<br>2. Balancing on a line<br>• Arms extended<br>• No arms<br>• Beanbag on head<br>3. Balancing with Dowels (practice steering)<br>4. Follow the Leader<br>5. Break into "Group 1"- able to handle bikes without assistance vs. "group 2" need assistance to get on and off<br>6. Pre-Test: "Count how long you can stride" (feet up- 3 tries)-record distance<br>7. Closing huddle	Stop watch<br>Cones<br>Tape to make lines or sidewalk chalk<br>6 bean bags<br>6 dowel rods<br>First aid kit<br>Camera<br>Notebook and pen to record length of striding<br>Name tags
DAY 2	1. Body warm up<br>2. Balancing on a line<br>3. Balancing with dowels<br>4. Dowel obstacle course<br>5. Froggie Races<br>6. Froggie Races with markers<br>7. Group 1: clockwise and counterclockwise turns<br>8. Group 2: Weaving through cones<br>9. Closing huddle	Cones<br>Tape to make lines or side walk chalk<br>Animal prints<br>6 dowel rods<br>First aid kit<br>Camera
DAY 3	1. Body warm up<br>2. Balancing with dowels	Cones<br>Tape to make lines or sidewalk chalk

	3. Dowel obstacle course<br>4. Froggie Races with markers<br>5. Group1: clockwise and counterclockwise turns<br>6. Group 2: Weaving through cones<br>7. High 5 game<br>8. Closing huddle	Animal prints<br>6 dowel rods<br>First aid kit<br>Camera
DAY 4	1. Body warm up<br>2. Group1: clockwise and counterclockwise turns<br>3. Group 2: Weaving through cones<br>4. Follow the leader (in 2 groups in necessary)<br>5. Red light Green light<br>6. Closing huddle	Cones<br>Tape to make lines or sidewalk chalk<br>6 dowel rods<br>First aid kit<br>Camera<br>STOP/ go sign
DAY 5	1. Body warm up<br>2. Group1: clockwise and counterclockwise turns<br>3. Group 2: Weaving through cones<br>4. Obstacle course<br>5. Post-Test: “Count how long you can stride” (feet up- 3 tries)-record distance<br>6. Awards<br>7. Closing huddle	Cones<br>Tape to make lines or sidewalk chalk<br>First aid kit<br>Camera<br>Number visuals (1-8)<br>Bubble wrap<br>Medals/awards<br>Parent Handouts<br>Notebook and pen
