# Supplementary material for: It’s Not Just about Bicycle Riding: Sensory-Motor, Social and Emotional Benefits for Children with and without Developmental Disabilities
Source: Children (Basel). 2022 Aug 13;9(8):1224. doi: 10.3390/children9081224 (PMC9406935; doi:10.3390/children9081224)
Supplement: Supplementary file 1 [file children-09-01224-s001.zip › Table S1.pdf]

Table S1. Study 1, Movement Activities, Mean Level and Slope

	Participant 1		Participant 2		Participant 3		Participant 4	
	BL<br>M<br>(Slope)	IN<br>M<br>(Slope)	BL<br>M<br>(Slope)	IN<br>M<br>(Slope)	BL<br>M<br>(Slope)	IN<br>M<br>(Slope)	BL<br>M<br>(Slope)	IN<br>M<br>(Slope)
Reach & Rock	3.33<br>(1.50)	4.50<br>(1.00)	0<br>(0)	1.25<br>(1.30)	0.33<br>(-0.50)	2.75<br>(0.90)	0<br>(0)	0.75<br>(0.90)
Standing Broad Jump	33.33<br>(2.00)	40.67<br>(0)	29.67<br>(0.50)	35.38<br>(0.75)	53.00<br>(0.50)	56.00<br>(1.00)	30.00<br>(4.50)	48.50<br>(3.00)
Romberg, Floor, Eyes Open	6.33<br>(-0.50)	7.67<br>(3.50)						
One Foot, Pad, Eyes Closed	1.67<br>(0.50)	2.33<br>(1.00)			4.67<br>(1.50)	7.00<br>(1.50)	1.00<br>(0)	2.50<br>(-0.40)
Romberg, Floor, Eyes Closed			4.67<br>(-1.50)	5.50<br>(2.60)				
Romberg, Pad, Eyes Open			5.67<br>(-3.00)	6.25<br>(3.10)	2.33<br>(-1.00)	8.25<br>(1.50)		
Heel-Toe Forward	4.33<br>(1.50)	7.33<br>(1.50)	5.33<br>(0)	5.33<br>(-0.50)	6.33<br>(2.50)	11.75<br>(0.10)	4.67<br>(0.50)	6.00<br>(-0.20)
Backwards Walking	5.00<br>(1.50)	2.67<br>(-0.50)	2.33<br>(0)	3.67<br>(1.50)	6.33<br>(0.50)	5.00<br>(-1.60)	3.67<br>(-2.00)	6.75<br>(0.10)

Note: BL = Baseline, IN = Intervention, M = Mean level
