# Supplementary figures and images for: It’s Not Just about Bicycle Riding: Sensory-Motor, Social and Emotional Benefits for Children with and without Developmental Disabilities
Source: Children (Basel). 2022 Aug 13;9(8):1224. doi: 10.3390/children9081224 (PMC9406935; doi:10.3390/children9081224)

Figure S1. Study 1 Participant 1

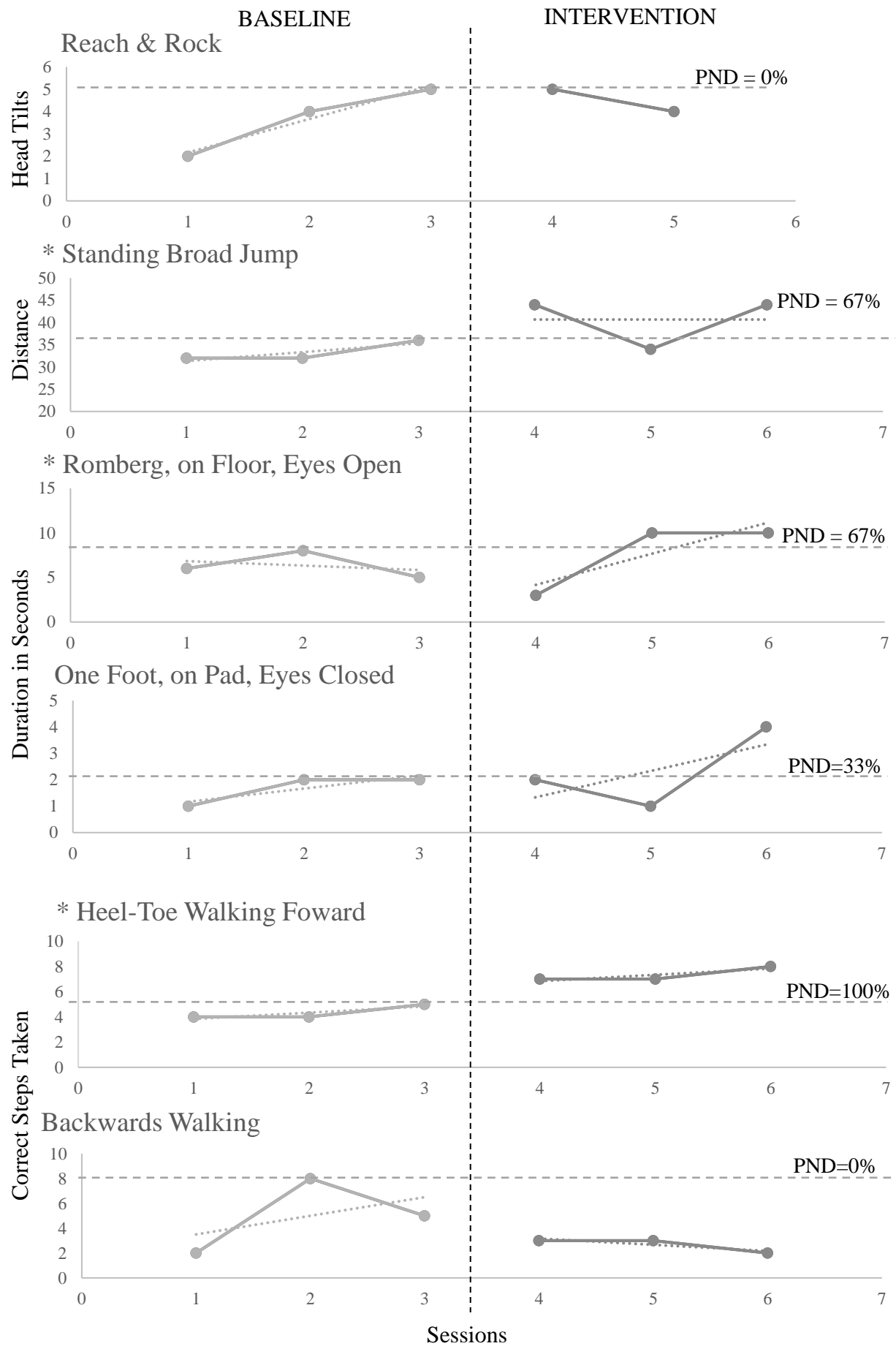

Supplement: Supplementary file 1 [file children-09-01224-s001.zip › Figure S1.pdf]

Figure S2. Study 1 Participant 2

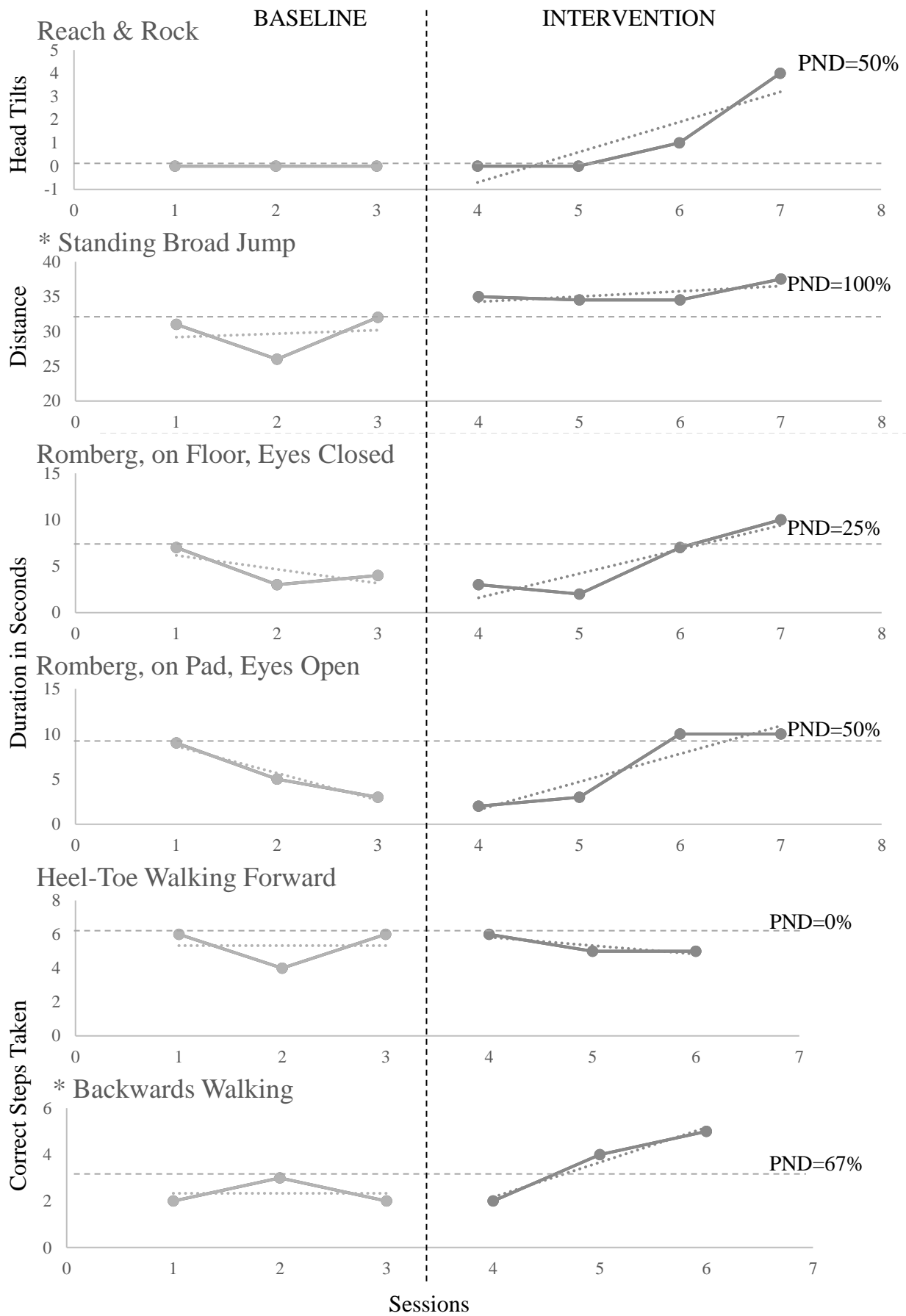

Supplement: Supplementary file 1 [file children-09-01224-s001.zip › Figure S2.pdf]

Figure S3. Study 1 Participant 3

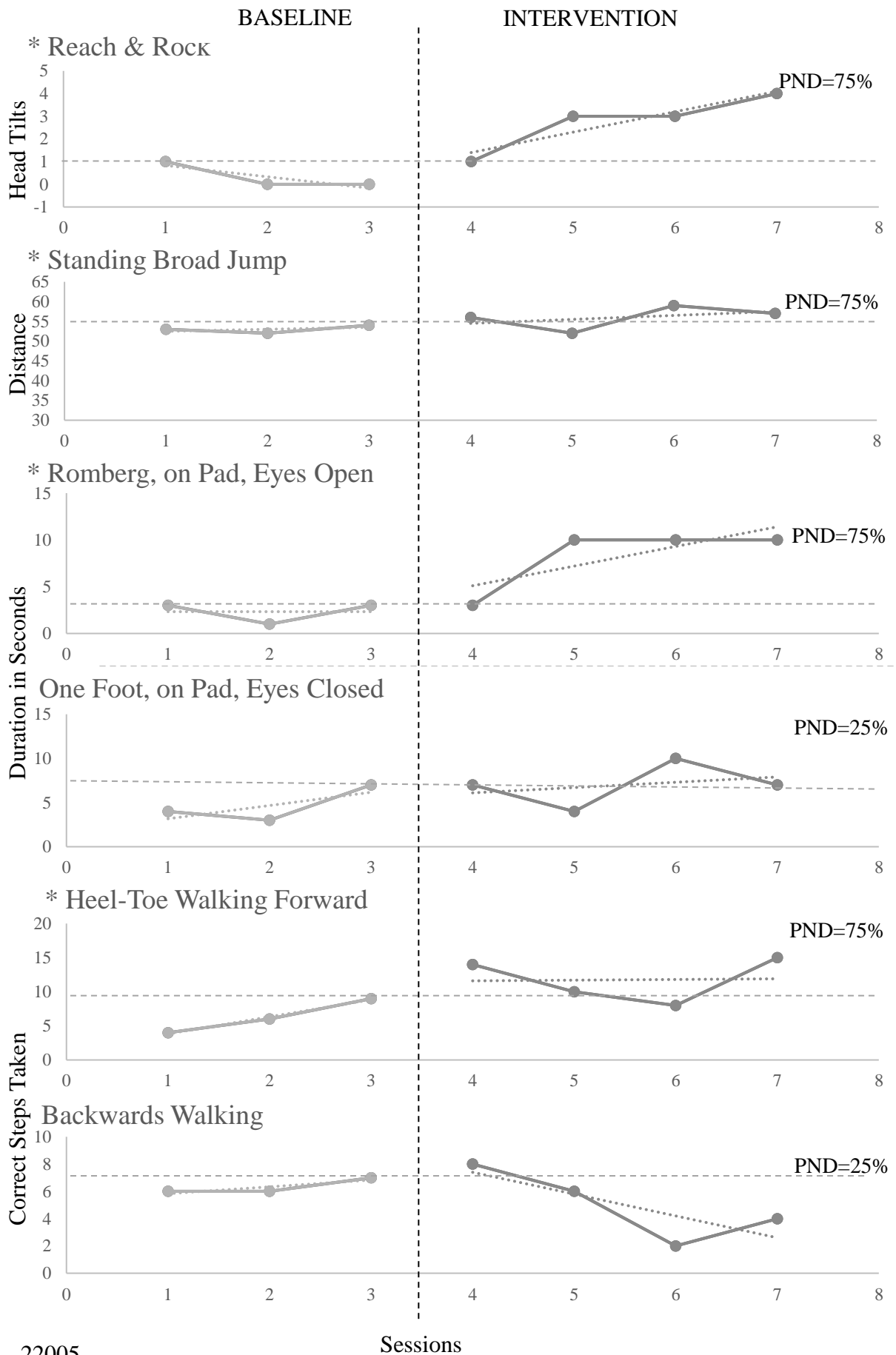

Supplement: Supplementary file 1 [file children-09-01224-s001.zip › Figure S3.pdf]

Figure S4. Study 1 Participant 4

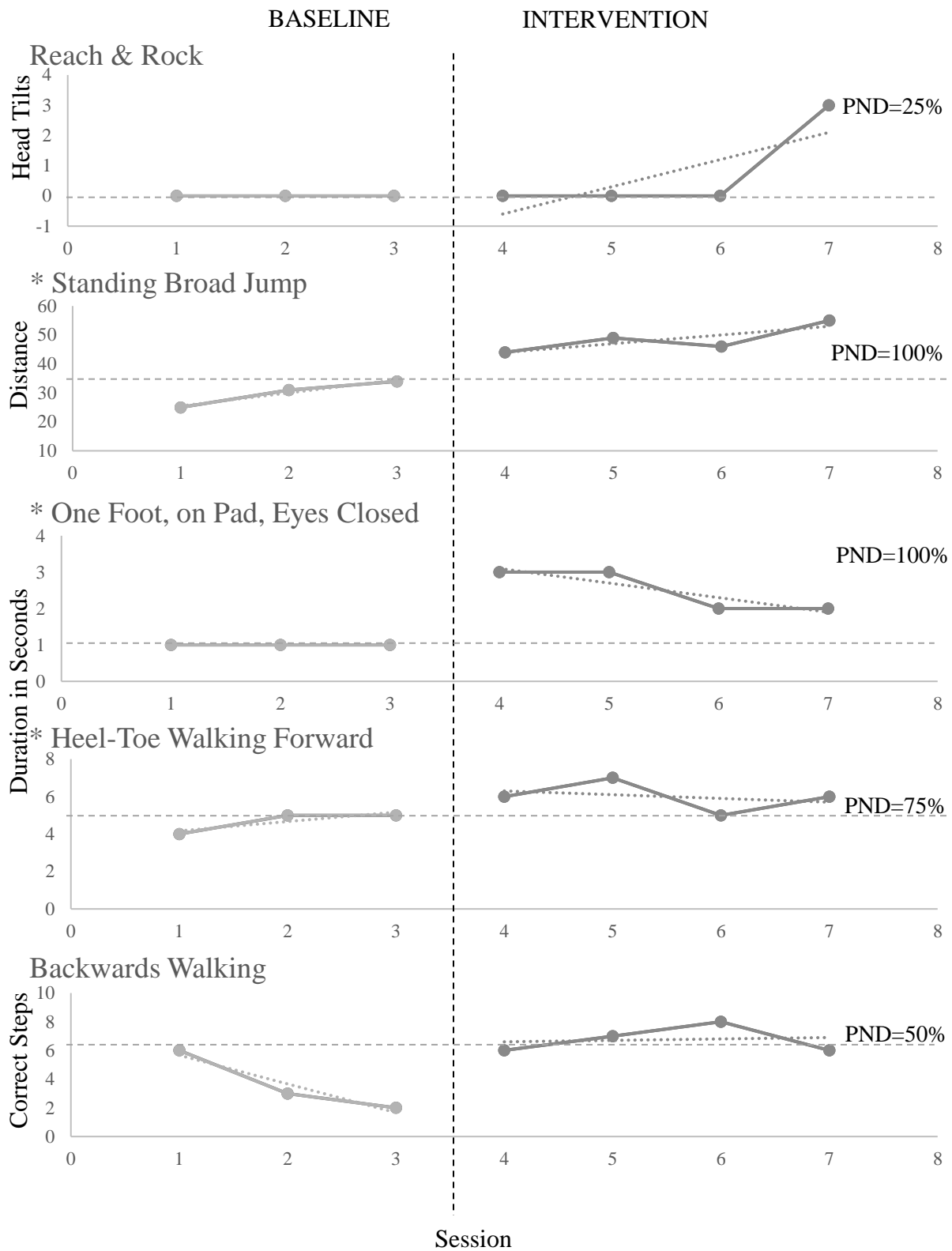

Supplement: Supplementary file 1 [file children-09-01224-s001.zip › Figure S4.pdf]
